# Vascular galectins in tumor angiogenesis and cancer immunity

**DOI:** 10.1007/s00281-024-01014-9

**Published:** 2024-07-11

**Authors:** Victor L. J. L. Thijssen

**Affiliations:** 1 Radiation Oncology, Amsterdam UMC Location Vrije Universiteit Amsterdam, De Boelelaan 1117, 1081 HV Amsterdam, Netherlands; 2 Center for Experimental and Molecular Medicine, Laboratory for Experimental Oncology and Radiobiology, Meibergdreef 9, 1105 AZ Amsterdam, the Netherlands; 3 Cancer Center Amsterdam, Cancer Biology & Immunology, Amsterdam, The Netherlands

**Keywords:** Endothelium, Tumor microenvironment, Immune cells, Immune check point, Glycosylation

## Abstract

Sustained tumor angiogenesis, i.e., the induction and maintenance of blood vessel growth by tumor cells, is one of the hallmarks of cancer. The vascularization of malignant tissues not only facilitates tumor growth and metastasis, but also contributes to immune evasion. Important players in all these processes are the endothelial cells which line the luminal side of blood vessel. In the tumor vasculature, these cells are actively involved in angiogenesis as well in the hampered recruitment of immune cells. This is the result of the abnormal tumor microenvironment which triggers both angiostimulatory and immune inhibitory gene expression profiles in endothelial cells. In recent years, it has become evident that galectins constitute a protein family that is expressed in the tumor endothelium. Moreover, several members of this glycan-binding protein family have been found to facilitate tumor angiogenesis and stimulate immune suppression. All this has identified galectins as potential therapeutic targets to simultaneously hamper tumor angiogenesis and alleviate immune suppression. The current review provides a brief introduction in the human galectin protein family. The current knowledge regarding the expression and regulation of galectins in endothelial cells is summarized. Furthermore, an overview of the role that endothelial galectins play in tumor angiogenesis and tumor immunomodulation is provided. Finally, some outstanding questions are discussed that should be addressed by future research efforts. This will help to fully understand the contribution of endothelial galectins to tumor progression and to exploit endothelial galectins for cancer therapy.

## Introduction

The human vasculature is a complex network of blood and lymphatic vessels that facilitate the transport of, amongst others, erythrocytes, leukocytes, thrombocytes and a wide variety of molecules. With a projected length of 100,000 km, the vasculature provides a huge infrastructural system that covers all parts of the human body. All vessels within the vasculature share one important common feature, i.e., their luminal side is composed of endothelial cells. These mesoderm-derived cells form a single cell layer, known as the endothelium, covering the entire inner surface of all vessel walls. It has been estimated that the endothelium comprises over one trillion (> 1 × 10^12^) endothelial cells, forming a surface area of 300–1000 square meters [1, 2]. This huge interface primarily serves as a barrier between cells and components in the blood and the underlying tissues. However, the endothelial barrier is far from inert and endothelial cells are known to be actively involved in many physiological processes, like regulation of the vascular tone, coagulation, inflammation, and bi-directional transport over the vessel wall [3–6]. In addition, endothelial cells are indispensable for angiogenesis, i.e., the formation of new blood vessels out of pre-existing vessels. This process is involved in physiological processes like wound healing, inflammation, the menstrual cycle, and pregnancy [3, 7, 8].

Given their widespread presence and diverse functionality, it is not surprising that abnormal endothelial cell activity and aberrant angiogenesis are also associated with different disorders, e.g., rheumatoid arthritis, atherosclerosis, inflammatory disorders, and malignant disease [3, 6, 9, 10]. Regarding the latter, the ability of tumor cells to induce and sustain angiogenesis is considered one of the hallmarks of cancer [11]. Already more than 50 years ago, it was found that most solid tumors become dependent on angiogenesis after reaching a volume 2–3 mm^3^ [12, 13]. At larger volumes, the high metabolic demand of tumor cells can no longer be fulfilled by diffusion of oxygen and nutrients from existing vessels. As a response, tumor cells start to secrete growth factors that activate endothelial cells in nearby vessels in order to trigger the growth of new blood vessels into the expanding tumor mass. Based on the observed dependency on angiogenesis it has been proposed that targeting tumor blood vessel growth could provide an opportunity for cancer therapy [14]. This insight boosted research into the mechanisms that control tumor angiogenesis and has resulted in the development of numerous angiostatic drugs that are currently used in the clinic [15, 16]. Unfortunately, current angiostatic drugs show only limited clinical benefit due to the development of treatment resistance [17, 18]. Thus, there is still a need to find novel targets and to develop more potent angiostatic drugs.

Over the last three decades, galectins have been identified as key regulators of endothelial cell function and tumor angiogenesis. Moreover, vascular galectins have been found to play a significant role in cancer progression, not only in the context of tumor angiogenesis, but also by suppressing an adequate anti-tumor immune response [19, 20]. This has identified galectins as potential targets for tumor therapy. The current review will first give a general introduction into the human galectin protein family and subsequently summarize the current knowledge regarding the role of vascular galectins in tumor angiogenesis and tumor immune escape.

## The galectin protein family

**Fig. 1:**
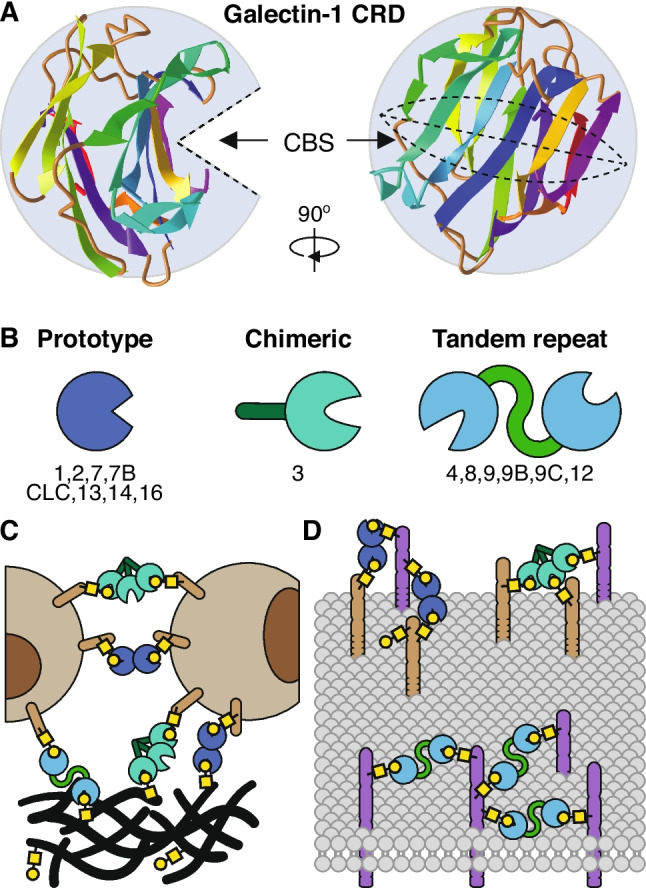
The galectin protein family. **A)** Graphical representation of the general structure of a galectin carbohydrate recognition domain (CRD, galectin-1 is used as example). Two antiparallel β-sheets (strands in different colors) form an overall β-sandwich which is curved, creating a 'groove' in which the core carbohydrate binding site (CBS) is located. Structural differences in the CRD and the binding groove contribute to the glycan-binding affinity and specificity of each galectin. Given its structure, the CRD is commonly depicted as a 'PacMan'-shape in which the CBS is represented as the 'mouth' that grabs the glycan moiety. **B)** Classification of the different galectin family members is based on the number and structural organization of the CRDs. The three subtypes include prototype galectins, chimeric galectins, and tandem repeat galectins. **C + D)** Extracellular activity of galectins is based on their glycan binding valency which can be increased by CRD dimerization or multimerization. The multivalency allows galectins to mediate interactions of cells with other cells or the extracellular matrix (C). In addition, multivalent galectins can facilitate (hetero)dimerization or clustering of cell surface receptors, thereby regulating receptor signaling (**D**)

Galectins (formerly known as S-type or S-Lac lectins) represent a subfamily of the animal lectin family [21] and they are defined by structural homology and binding affinity towards β-galactose-containing glycoconjugates [22]. The glycan-binding activity resides in a carbohydrate binding site (CBS) which is located in an evolutionary conserved carbohydrate-recognition domain (CRD) [23]. Generally, the galectin CRD, comprises 120–140 amino acids that fold into a β-sandwich structure consisting of two antiparallel β-sheets of six (S1–S6) and five (F1–F5) β-strands. The overall β-sandwich is curved, creating a 'groove' on the concave side which forms the CBS [24]. The core β-galactose binding occurs within this CBS but structural differences in the CRD and the binding groove further contribute to the glycan-binding affinity and specificity of each galectin (Fig. 1A) [24–26]. It is also important to realize that each galectin can bind different glycoconjugates with affinities ranging from millimolar down to nanomolar. This variable affinity depends on glycan composition and complexity [27] but also on the biological context of the glycoconjugate, e.g., in solution or on the cell surface [26]. Moreover, different galectins are subjected to posttranscriptional and posttranslational modifications, like splicing, phosphorylation, and proteolytic cleavage, which can affect their glycan binding and control their biological availability and activity [28–33]. Finally, recent studies have shown that the glycan binding can also be altered by peptides and proteins that interact with galectins thereby inducing structural changes in the CRD [34–36]. All these findings show that the galectin CRD is not a rigid structure but that it is capable to interact with different glycoconjugates as well as non-glycosylated proteins.

In humans, 15 galectins have been described so far and galectins are generally classified into three subtypes based on the number and organization of the CRDs, (Table 1 + Fig. 1B), i.e., i) prototypical galectins, which consist of a single CRD, ii) tandem-repeat galectins, which have two distinct CRDs connected by a short linker domain, and iii) chimeric galectins, of which only 1 member has been described so far which is characterized by a unique N-terminal domain linked to a single CRD. Galectin functionality is diverse as most galectins can be found both intracellularly as well as in the extracellular environment [25]. However, a key aspect of galectin functionality resides in their ability to dimerize or multimerize. This complex formation increases the binding valency and allows galectins to form 'networks' within or between glycoconjugates [37–39]. Consequently, in the extracellular environment galectins can facilitate cell–cell interactions and contribute to cell–matrix adhesion as well as migration (Fig. 1C). Moreover, by clustering cell surface receptors and/or increase receptor surface retention, galectins can modulate signaling (Fig. 1D) [38, 40]. Intracellularly, galectins can also enhance receptor signaling, expedite secretion of cytokines or recognize cytosolic glycoconjugates. Even in the nucleus, galectins have been found to be actively involved in RNA splicing [41–43]. It is important to realize that these examples are just a selection of known galectin activities and ongoing research continues to discover new functionalities of this versatile protein family. The current review will further focus on the expression of galectins in the endothelium and their role in tumor angiogenesis and tumor immune evasion.

**Table 1:** The human galectin protein family^a^

Name	Subtype^b^	Symbol	Alias symbols	Location	Ensembl gene ID
galectin 1	P	LGALS1	GBP	22q13.1	ENSG00000100097
galectin 2	P	LGALS2	HL14	22q13.1	ENSG00000100079
galectin 3	C	LGALS3	MAC-2, GALIG	14q22.3	ENSG00000131981
galectin 4	TR	LGALS4	GAL4	19q13.2	ENSG00000171747
galectin 7	P	LGALS7	GAL7/PIG1/TP53I1/LGALS7A	19q13.13	ENSG00000205076
galectin 7B	P	LGALS7B	GAL7	19q13.2	ENSG00000178934
galectin 8	TR	LGALS8	PCTA-1	1q43	ENSG00000116977
galectin 9	TR	LGALS9	LGALS9A	17q11.2	ENSG00000168961
galectin 9B	TR	LGALS9B		17p11.2	ENSG00000170298
galectin 9C	TR	LGALS9C		17p11.2	ENSG00000171916
Charcot-Leyden crystal galectin	P	CLC	LGALS10/MGC149659/Gal-10	19q13.2	ENSG00000105205
galectin 12	TR	LGALS12	GRIP1	11q12.3	ENSG00000133317
galectin 13	P	LGALS13	PP13, PLAC8	19q13.2	ENSG00000105198
galectin 14	P	LGALS14	PPL13, CLC2	19q13.2	ENSG00000006659
galectin 16	P	LGALS16		19q13.2	ENSG00000249861

a) Approved nomenclature obtained from the HGNC (HUGO Gene Nomenclature Committee) [161]

b) P = prototype; c = Chimeric; TR = tandem repeat

## Endothelial galectin expression

**Fig. 2:**
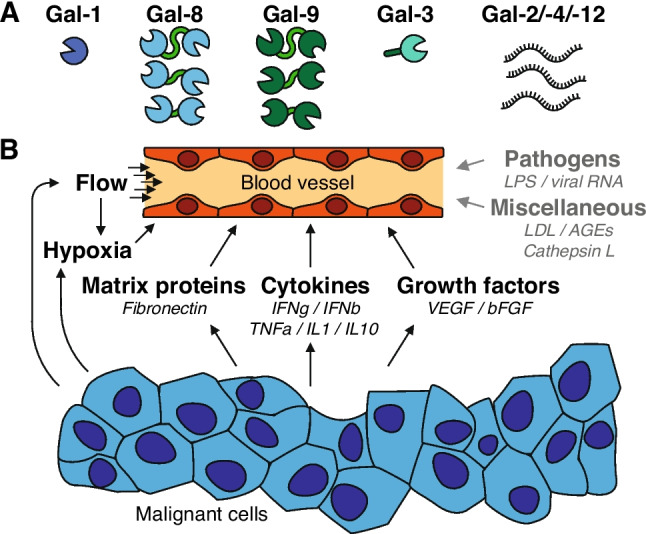
Endothelial galectin expression and regulation. **A)** Endothelial cells express 4 galectins at the protein level, i.e., galectin-1/-3/-8/-9. These galectins can be found both intracellularly or on the endothelial cell surface. In addition, different splice variants of galectin-8 and galectin-9 have been detected in endothelial cells. Three galectins, i.e., galectin-2/-4/-12, have been detected at the mRNA level but expression at the protein level awaits confirmation. **B)** Schematic representation of the different environmental conditions that have been shown to affect the galectin expression and/or localization in endothelial cells. See the main text for more details. Note that many of the environmental triggers are also frequently found in tumor tissues. Non-malignant triggers are shown in grey. IFNg = Interferon gamma; IFNb = Interferon beta; TNFa = Tumor Necrosis Factor alpha; IL = Interleukin; LPS = Lipopolysaccharides; LDL = low-density lipoprotein; AGEs = Advanced glycation end-products

It is well established that multiple galectins are expressed by endothelial cells. In general, out of the 15 galectins found in humans, endothelial cells predominantly express galectin-1, -3, -8, and -9 [44, 45]. Of the latter two, different splice variants can be detected in endothelial cells [44, 46–48]. Of the other galectins, only galectin-2, -4, and -12 show some expression at the mRNA level but no detectable protein levels, while the expression of the remaining galectins appears to be completely absent (Fig. 2A) [44]. At the same time, the endothelial expression of galectins is highly variable and depends on different factors, including the specific endothelial cell type, the tissue location as well as environmental conditions. For example, it has been reported that lymphatic endothelial cells show a relatively high expression of galectin-8 [46] while endothelial progenitor cells express relatively high levels of galectin-3 [49]. Such differences are likely related to the environmental conditions in which the endothelial cells reside. For example, irregular blood flow has been shown to trigger endothelial expression of galectin-3 [50]. In line with this, hypoxic conditions have been shown to induce galectin-3 expression in endothelial cells [51]. Other known triggers that affect the expression and/or secretion of galectins by endothelial cells include matrix components, like fibronectin or advanced glycosylation end products [52, 53], inflammatory cytokines like INFγ and TNFα as well as growth factors that induce endothelial activation, like vascular endothelial growth factor (VEGF) (Summarized in Fig. 2B + Table 2) [33, 44, 54–56]. Gaining further insight in the regulation of endothelial galectin expression, in particular in the context of the complex tissue microenvironment, remains an ongoing challenge.

**Table 2:** Factors that regulate endothelial galectin expression

Cell type	Galectin	Stimulatory factor	Inhibitory factor	References
HUVEC	galectin-1	20% HS (i), TCM		[44, 55, 59, 113]
HOMEC	galectin-1	Cathepsin-L		[162]
Ea.hy926 cells	galectin-1		Neutrophil transmigration (iii)	[163]
HUVEC	galectin-3	Turbulant flow, IL-1b		[44, 50, 55, 125]
EPC	galectin-3	higher expression in EPC vs. HUVEC/LMEC/AoEC		[49]
PAEC	galectin-3	Hypoxia		[51]
EPC	galectin-3	Fibronectin		[52]
HMEC-1	galectin-3	AGEs		[53]
Ea.hy926 cells	galectin-3	Neutrophil transmigration (iv)		[163]
HUVEC	galectin-8		20% HS (i)	[44]
PLEC	galectin-8	Higher expression in LEC vs BEC		[46]
HMEC-1	galectin-8	LPS		[130]
HUVEC	galectin-9	IFNg, IFNb, Poly I:C, IL-10, viral RNA	20% HS (i, ii), VEGF, IL-1	[33, 44, 54-56, 164]

(i) No additional effect of bFGF or tumor conditioned medium; (ii) Overal inhibition, but translocation to cell surface; (iii) Only significantly reduced in nuleus; (iv) Only significantly increased in cytoplasm and on cell surface; HUVEC = Human umbilical vein endothelial cells; HS = Human serum; TCM = Tumor-conditioned medium; HOMEC = Human omental microvascular endothelial cells; Ea.hy926 cells = HUVEC:epithelioma A549-hybridoma; PLEC = Primary lymphatic endothelial cells; PBVEC = Primary blood vessel endothelial cells; PAEC = Pulmonary artery endothelial cells; EPC = Endothelial progenitar cells; HMEC-1 = Human microvascular endothelial cell line; LMEC = Human lung microvascular endothelial cells; AoEC = Human aortic endothelial cells; IFNg = Interferon gamma; IFNb = Interferon beta; TNFa = Tumor Necrosis Factor alpha; IL = Interleukin; LPS = Lipopolysaccharides; LDL = low-density lipoprotein; AGEs = Advanced glycation end-products; VEGF = Vascular endothelial growth factor;

Given the abnormal microenvironmental conditions in malignant tissues, it is not surprising that the tumor endothelium displays altered galectin expression compared to normal endothelium. For example, we reported on elevated galectin-1 expression in tumor endothelium vs. normal endothelium in colon carcinoma, breast carcinoma and sarcoma [57]. Others found increased galectin-1 expression in tumor endothelium from oral squamous cell carcinoma [58], prostate cancer [59], lung cancer and head and neck cancer [60]. Likewise, endothelial galectin-3 expression has also been reported in different tumor tissues, e.g., lung [60], head and neck [60], colon [44], and primary central nervous system lymphomas [61]. In the latter, increased endothelial galectin-3 expression was associated with poor patient survival [61]. On the other hand, endothelial galectin-3 expression levels appear to decrease with increasing malignancy in brain tumors [62–64]. Moreover, patients with low endothelial galectin-3 expression in primary oligodendrogliomas and anaplastic oligodendrogliomas had significantly shorter progression free and overall survival [63].

With regard to galectin-8, we and others reported that cultured endothelial cells express 3 splice variants [44, 47]. Moreover, activation of cultured endothelial cells by high serum conditions induced a decrease in galectin-8 expression [44]. While this was not further affected by tumor-derived conditioned medium, we did observe an additional reduction in cell surface galectin-8 on endothelial cells after conditioned medium treatment [44]. Of note, in vivo endothelial expression of galectin-8 was only observed in the vasculature of normal colon tissues and sporadically in kidney. When detected, the protein was mainly localized in the nuclei of endothelial cells. In addition, in colon tumor tissues, the frequency of galectin-8 positive endothelial cells was even reduced [44]. Delgado and colleagues also observed clear nuclear galectin-8 expression in blood vessels of both normal and malignant tissues from prostate and breast. Endothelial cells in the prostate also showed cytoplasmic galectin-8 expression, while this was more diffuse in breast tissues [47].

Similar as for galectin-8, endothelial cells have been reported to express different splice variants of galectin-9 [44, 65]. Moreover, the expression and localization of galectin-9 also appears to be regulated by endothelial cell activation status and/or different inflammatory conditions [44, 54, 66]. Endothelial expression of galectin-9 has also been reported to be increased in the tumor vasculature. For example, we observed significantly elevated galectin-9 expression in the tumor endothelium of lung, liver and kidney cancer compared to healthy tissues [31].

Overall, endothelial cells predominantly express galectin-1, -3, -8, and -9. Moreover, in tumor tissues the endothelial expression level and/or localization of these galectins is often different as compared to endothelial cells in normal tissues. This different expression is predominantly triggered by the abnormal tumor microenvironment. Factors that regulate endothelial galectin expression include matrix proteins, growth factors and inflammatory cytokines but also flow and hypoxia. Despite the increasing insights in the regulation of endothelial galectin expression it remains a future challenge to unravel the exact interplay between the tumor microenvironment and galectin expression. Especially since environmental conditions not only influence galectin expression but also affect the expression and composition of glycoconjugates on the endothelial cell surface [67–71]. Moreover, it can be anticipated that the complex regulation of vascular galectins and their ligands by the tumor microenvironment contributes to tumor progression.

## Endothelial galectins in tumor progression

As evident from the above, the tumor endothelium is characterized by altered galectin expression. Interestingly, abnormal galectin expression, be it in tumor cells and/or tumor associated stroma, is associated with the diagnosis and/or prognosis of patients with different cancer types [72]. Based on this association, extensive research has been performed to uncover the mechanisms by which galectins contribute to malignant transformation and tumor progression. This has revealed that galectins contribute to most, if not all, hallmarks of cancer [11, 73, 74]. With regard to galectins in the vasculature, research has uncovered different mechanisms by which galectins in the endothelium contribute to tumor progression, including promoting tumor angiogenesis and suppressing the anti-tumor immune response.

### Role of vascular galectins in tumor angiogenesis

**Fig. 3:**
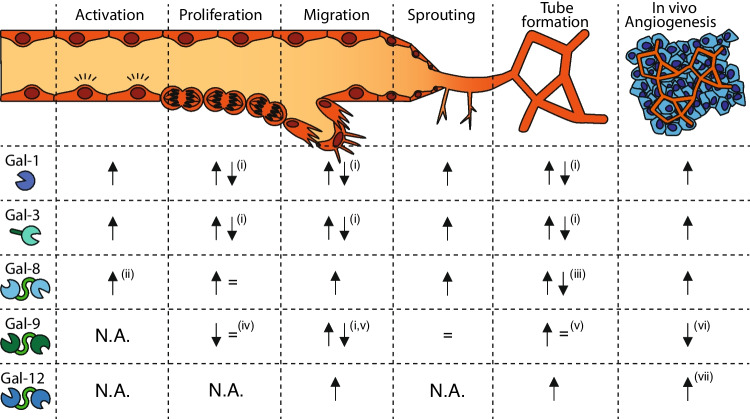
The role of endothelial galectins in angiogenesis. Schematic overview of the contribution of galectins to different steps in endothelial cell function in vitro and angiogenesis in vivo (Adapted and updated from [45]). See main text for more detail. It is important to note that the observed effects are dependent on the source of endothelial cells, the experimental conditions, and the source of galectin, e.g., extrinsic, intrinsic, recombinant. In addition, not all effects have been identified in the context of malignant disease. ↑ = stimulation; ↓ = inhibition; = no effect. (i) Concentration dependent, i.e., stimulation in low nM range and inhibition in high nM/low μM range; (ii) Inflammatory activation; (iii) Dependent on lymph (↓) vs. blood ( =) endothelial cell phenotype; iv) Different effects were found for the individual domains of galectin-9 [33]; (v) Dependent on cell activation status; (vi) Only at high dose (500 nM); (vii) Reduced adipose tissue vascularization in knockout mice. N.A. = Not analyzed

To understand the role of vascular galectins during tumor angiogenesis, it is important to realize that vessel growth, both under physiological or pathophysiological conditions, is a multistep process during which endothelial cells display different functionalities and behavior. In brief, endothelial cells have to become activated by stimulatory factors that initiate the angiogenic cascade. Once activated, endothelial cells start to remodel the extracellular environment and migrate towards the origin of stimulation. At the same time, cells have to proliferate and organize into tube-like structures in order to form new vessels. All these activities require endothelial cells to interact with the extracellular microenvironment and with other (endothelial) cells. Furthermore, proper execution of these steps involves a concerted action of many different signaling pathways that control the expression and activity of different proteins. Galectins have been identified as one of the key protein families that are involved in angiogenesis by mediating different endothelial cell functions. An overview of their role in angiogenesis was recently presented and is summarized in Fig. 3 [45]. Although not all of the angioregulatory activities of galectins have been identified in the context of malignant disease, it can be anticipated that vascular galectins exert comparable effects on endothelial function during tumor angiogenesis. In line with this, we were the first to show that galectin-1 is essential for tumor vascularization, independent of the tumor type [57, 75]. Furthermore, we and others found that endothelial cells can also exploit extrinsic galectin-1 that is secreted by tumor cells [74–76] or cancer associated fibroblasts to increase their angiogenic potential [77].

A key role of galectin-1 in tumor angiogenesis is linked to activation of pro-angiogenic signaling by the vascular endothelial growth factor (VEGF). For example, galectin-1 was described to increase VEGF receptor 2 (VEGFR2) signaling in endothelial cells by facilitating heterodimerization with co-receptor neuropilin-1 (NRP1) [58]. In addition, galectin-1, in combination with galectin-3, was suggested to hamper the internalization of VEGFR1/2 and thereby increasing receptor signaling [78]. Galectin-1 was also linked to VEGFR1/NRP1 complex formation which contributes to vascular permeability in tumors [79]. Compelling evidence linking galectin-1 to VEGFR signaling in tumors was provided by Croci and co-workers. They identified a direct link between VEGFR2 glycosylation and the ability of galectin-1 to activate VEGFR2 signaling. In murine tumors that were non-responsive to anti-VEGF treatment, the altered VEGFR2 glycosylation allowed galectin-1 to activate VEGF-like signaling which contributed to treatment escape [67]. These results also exemplified the important link between endothelial cell glycosylation and their sensitivity to galectins, a link that requires further exploration [68, 76].

Apart from the prominent role in VEGF/VEGFR signaling, ample studies have linked galectin-1 to tumor progression by increasing endothelial cell proliferation and migration, thus facilitating endothelial cell sprouting, tube formation and tumor vascularization [20, 76, 80, 81].

Similar to galectin-1, pro-angiogenic activity has been attributed to galectin-3. As briefly mentioned above, galectin-3 was also found to facilitate VEGFR signaling in endothelial cells [82]. In addition, galectin-3 was found to interact with endoglin, a co-receptor of TGF beta signaling, which is involved in angiogenesis [83]. Galectin-3 also appears to increase endothelial cell adhesion, motility and migration by interacting with different integrins on the cell surface [84–86]. In models of breast and pancreas cancer, galectin-3 was shown to trigger endothelial cell functionality in vitro and stimulate tumor angiogenesis in vivo [87–89]. Furthermore, part of the angiostimulatory activity of galectin-3 has been linked to the modulation of Notch signaling. It was found that galectin-3 interacts with the Notch ligand JAG1, thereby increasing the half-life of the protein and activating endothelial JAG1/Notch-1 signaling. Consequently, tumor growth in a murine lung tumor model was increased when JAG1 overexpressing tumor cells were used and hampered in galectin-3 knockout mice [69]. Of note, it has been proposed that the angiogenic activity of galectin-3, at least in breast cancer, is dependent on proteolytic cleavage of the galectin-3 N-terminal tail [90]. To what extent full length and cleaved galectin-3 trigger similar or distinct angiostimulatory pathways should be further explored.

More recently, galectin-8 was added to the list of galectins that facilitate VEGF-induced angiogenesis both in vitro and in vivo [91]. Previously, galectin-8 was already found to stimulate endothelial cell migration and tube formation in vitro and angiogenesis in vivo [47]. This activity was linked to interactions of galectin-8 with CD166 (ALCAM; activated leukocyte cell adhesion molecule) [47]. At the same time, galectin-8 was described to interact with different integrins expressed by endothelial cells [92] which could further add to the pro-angiogenic activity. Galectin-8 secreted by tumor cells was also found to induce vascular permeabilization [93], one of the first steps in the endothelial response to pro-angiogenic stimulation [94]. With regard to tumor angiogenesis, it has been described that elevated galectin-8 serum levels, which were observed in breast and colorectal cancer [95], can stimulate endothelial cell tube formation [96]. Thus, similar to galectin-1 and galectin-3, current findings suggests that galectin-8 can stimulate endothelial cell functions but identifying the exact role of the protein in tumor angiogenesis requires further investigation.

With regard to the role of endothelial galectin-9 in tumor angiogenesis also little information is available. As already described above, the tumor endothelium in different cancer types showed elevated galectin-9 expression [31]. However, to what extent the protein contributes to tumor vessel formation is unknown. For example, whether the protein facilitates VEGF/VEGFR signaling -similar as the other endothelial galectins- has not been described. Galectin-9 was found to serve as a chemoattractant for endothelial cells and to stimulate migration and tube formation [33, 97]. At the same time, the effects of galectin-9 appear to be dependent on the activation status of the endothelial cells as well as on the protein isoform and concentration [33]. Regarding the latter, we observed a slight inhibition of in vivo angiogenesis in the chicken chorioallantoic membrane model by galectin-9 at micromolar concentrations while in vitro stimulatory effects were observed at nanomolar concentrations [33]. Since most studies were performed using in vitro models or in the context of developmental angiogenesis, more research is required to further unravel the contribution of endothelial galectin-9 to tumor angiogenesis. The same applies to galectin-12, the latest addition to the list of angioregulatory galectins. Although -again- no information is available in the context of tumor angiogenesis, it was observed that exogenous galectin-12 from adipose tissue can trigger the angiogenic activity of endothelial cells in vitro and in vivo. This was linked to increased availability of fucosylated glycan ligands on endothelial cells under hypoxic conditions [70]. Whether the same mechanism is active in the hypoxic tumor microenvironment awaits further studies. Furthermore, since galectin-12 is not expressed by endothelial cells, these findings warrant investigation into the angioregulatory activity of other galectins that are not expressed by endothelial cells but that do show effects on endothelial cell function, e.g., galectin-2 and galectin-4 [96].

### Role of endothelial galectins in tumor immunity

**Fig. 4:**
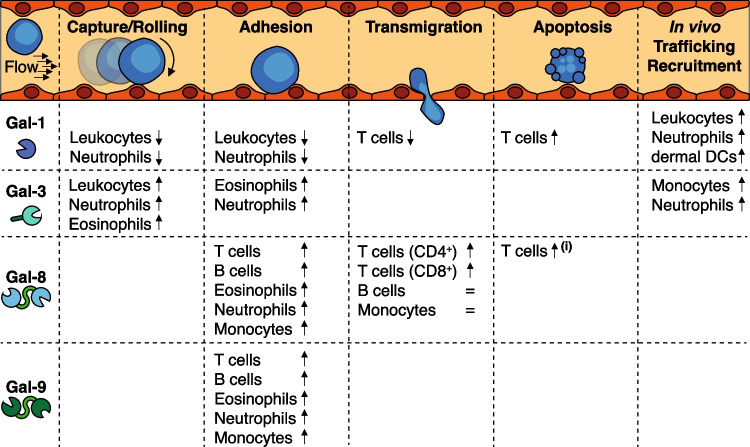
The role of endothelial galectins in immunomodulation. Schematic overview of the contribution of endothelial galectins on the different steps in immune cell recruitment via the endothelium. See main text for more detail. It is important to note that not all activity shown here was studied in the context of tumor angiogenesis. This is relevant since the tumor endothelium is known to reduce the expression of endothelial adhesion molecules that are normally induced under inflammatory conditions. Thus, not all of the galectin activities shown here might occur in the tumor endothelium. At the same time, tumor-derived galectins are known to affect immune cell function and survival, e.g., T cell apoptosis induction by galectin-9 or amplification of B cell activation by galectin-1 (see [107] for comprehensive review). The current overview only lists effects related to endothelial galectins. As evident from the overview, there is still a lot of information lacking which warrants further research. ↑ = stimulation; ↓ = inhibition; = no effect. (i) Only in activated T cells. In naïve T cells, galectin-8 indirectly stimulates proliferation

The previous paragraphs summarized how galectins can contribute to tumor progression by regulating endothelial cell activity and tumor angiogenesis. Interestingly, tumor angiogenesis is linked to another hallmark of cancer, i.e., avoiding immune destruction. Indeed, different mechanisms have been described by which angiogenesis, or activated endothelial cells, contribute to immunosuppression, e.g., decreased endothelial expression of leukocyte adhesion molecules as well as increased endothelial expression of immune checkpoint molecules (for excellent recent reviews, see [98, 99]). These mechanisms create a so-called immune-cell barrier at the blood-endothelium interface, and galectins contribute to this barrier by affecting the survival and trafficking of immune cells in the tumor microenvironment. As such, endothelial galectins can be considered as family of proteins that link tumor angiogenesis to tumor immunosuppression [100]. Regarding the immunosuppression, it is important to realize that there is ample evidence of the immunoregulatory activity of galectins in different (patho)physiological processes [73, 101–106]. However, not being the scope of the current review to describe these activities in detail, it suffices to state that galectins can trigger a broad range of effects on both lymphoid and myeloid cells by interacting with specific glycoconjugates on the surface of these immune cells. Known effects include -but are not restricted to- recruitment, activation, differentiation, survival of, e.g., B cells, NK cells, regulatory and cytotoxic T cells, dendritic cells, granulocytes, monocytes and macrophages (For comprehensive overviews, see [107, 108]). In general, aberrant galectin expression in tumor cells and/or the tumor microenvironment is considered to contribute to tumor immune escape [105]. In line with this, different mechanisms have been identified by which endothelial galectins might contribute to immunomodulation, albeit not always in the context of malignant disease (Fig. 4) [109, 110]. For example, galectin-1 on the endothelial cell surface has been found to reduce the capture, rolling and adhesion of leukocytes as well as neutrophils on endothelial cells [111, 112]. In addition, endothelial galectin-1 was described to induce apoptosis and hamper transendothelial migration of activated T cells [113, 114]. The latter was linked to clustering of CD43 on T cells thereby interfering with the proper signaling required for migration [113]. In contrast, galectin-1 was found to trigger the migration of dendritic cells by facilitating co-cluster formation of CD43/CD45 [115]. In neutrophils, CD43-galectin-1 interactions as well as galectin-1 induced changes in L-selectin and beta-2 integrin have also been linked to increased neutrophil recruitment and migration [116, 117]. Moreover, galectin-1 stimulated the extravasation of polymorphonuclear leukocytes following lung injury by mediating interactions with endothelial cells [118]. Collectively and comparable to its role in facilitating VEGF receptor signaling during angiogenesis, galectin-1 appears to play an important role in regulating CD43/CD45 functionality, thereby controlling immune cell survival, migration and recruitment. At the same time, it has become evident that these regulatory effects vary between immune cell types and that they are dependent on specific environmental conditions. Thus, while additional research is still warranted, elevated galectin-1 expression in tumor endothelial cells appears to predominantly exert immunosuppressive effects.

The role of endothelial galectin-3 in tumor immunomodulation is still not fully understood but it appears that this family member acts somewhat more ambiguous as compared to galectin-1. In general, galectin-3 within the tumor micro-environment is considered to contribute to immunosuppression [119]. Similar as galectin-1, extracellular galectin-3 has been shown to hamper T cell survival and function, albeit that the exact T cell surface receptors underlying these responses, amongst others, CD7/CD29/CD43/CD45, are still not completely resolved [120, 121]. The interaction between galectin-3 and LAG3 (lymphocyte activation gene 3, CD223) has also been suggested to suppress effector CD8 + T cell function [122] but this also requires further confirmation [123]. Furthermore, whether endothelial cell derived galectin-3 exerts all these immunosuppressive functions at the blood-endothelial interface has not been confirmed yet. What has been described is that galectin-3 mediates the adhesion of neutrophils to endothelial cells. This appears to involve interactions of galectin-3 with ligands on both the neutrophil and the endothelial cell surface [124]. The same appears true for eosinophil-endothelial cell interactions as blocking antibodies that target galectin-3 on either endothelial cells or eosinophils were found to reduce the adhesion and rolling of the latter [125]. More recently, studies in galectin-3 knockout mice confirmed the importance of galectin-3 in decreasing leukocyte rolling and increasing recruitment of neutrophils and monocytes. The latter was linked to induction of an inflammatory environment by exogenous galectin-3 [126]. Since galectin-3 has been suggested to serve as a chemoattractant for monocytes and macrophages [127] it can be speculated that endothelial galectin-3 favors the recruitment and survival of myeloid cells over lymphoid cells. However, the exact contribution of endothelial galectin-3 to tumor immunomodulation still requires further research.

The tandem repeat galectin-8 has also been described to increase leukocyte-endothelial adhesion. In vitro assays revealed enhanced binding of T-cells, B-cells, neutrophils, eosinophils as well as monocytes to HUVEC in the presence of each galectin [128]. The same study also found some adhesion in the presence of either galectin-1 or galectin-3 but the effects were much less pronounced if detected at all. The discrepancies with the previously described roles of both galectin-1 and galectin-3 in leukocyte adhesion are most likely related to the variable experimental conditions regarding the galectin concentration and type of leukocyte or endothelial cells used. In particular, the presence of integrin beta1 appears to be required to facilitate the pro-adhesive function of galectin-8 [128, 129]. Of note, vascular adhesion of multiple myeloma cells (malignant cells of B cell origin) was also enhanced by galectin-8 [48]. Thus, galectin-8 appears to be involved in leukocyte trafficking. Next to this activity, galectin-8 was also found to induce direct proinflammatory effects by inducing the expression of proinflammatory cytokines in endothelial cells [130], activating dendritic cells [131] and increasing T cell proliferation [132]. However, the latter was only observed for naïve T cells while galectin-8 induced cell death of activated T cells [132], in line with the other galectins.

Galectin-9, the other tandem repeat galectin that is expressed by endothelial cells, has also been linked to increased leukocyte-endothelial adhesion. This includes similar populations as those linked to galectin-8-mediated adhesion, e.g., T cells, B cells, neutrophils and monocytes [128]. In addition, recent studies have provided additional insight in the specific activity of galectin-9 in leukocyte-endothelial interactions. For example, galectin-9, but not galectin-1 or -3, was shown to increase the adhesion of B cells to endothelial cells. However, the trans-endothelial migration was not affected and galectin-9 was found to trigger signaling indicative of B cell anergy [55]. The adhesion of CD14 + monocytes to activated endothelial cells (poly I:C stimulation) was also facilitated by galectin-9 while the transmigration was not [56]. In addition, the galectin-9 mediated monocyte adhesion appeared to be dependent on integrin beta2 (CD18) [56], an interaction that was also linked to the endothelial capture and adhesion of neutrophils and T cells [133, 134]. Regarding the latter, galectin-9 was reported to facilitate the adhesion to and migration over stimulated endothelial cells (TNF-α/IFN-γ) of both CD4 + and CD8 + T cells. Consequently, in an in vivo model of leukocyte recruitment (dorsal air pouch model), galectin-9 knockout mice showed hampered leukocyte trafficking [134]. Of note, it has also been suggested that Jurkat cells (immortalized human T cells) use galectin-9 for adhesion by interacting with blood group H glycans on the endothelial cell surface [135]. In addition, galectin-9 was reported to interact with protein disulfide isomerase on the T cell surface which promotes cell migration [136, 137]. Apparently, galectin-9 contributes to different mechanisms that regulate the recruitment and trafficking of leukocytes in the vasculature.

While the observed activities indicate an immunostimulatory function there is increasing interest in the immunosuppressive activity of galectin-9, in particular in the context of tumor immune escape. The immunosuppressive function is partly related to the ability of galectin-9 to bind TIM3 (T cell immunoglobulin and mucin domain containing protein 3) which is presented on the surface of different immune cells. The TIM-3/galectin-9 interaction alters TIM-3 signaling leading to immune cell anergy or apoptosis (for a recent review, see [138]). As such, the TIM3/galectin-9 signaling axis shows similarities with more 'classic' immune checkpoints like PD1/PD-L1 or CTLA-4/CD80 [138, 139]. Interestingly, it was shown that galectin-9 can also interact with PD-1 thereby attenuating galectin-9/TIM-3-induced apoptosis of exhausted CD8 + T cells in tumors [104]. Moreover, galectin-1 has been shown to induce tumor immune exclusion by increasing the endothelial expression of checkpoint molecules like PD-L1 as well as galectin-9 [140]. This supports the concept that endothelial galectins jointly act in creating an immunosuppressive environment. In line with this, galectins have been proposed to serve as immune checkpoint proteins that can be targeted for treatment of cancer [139].

## Summary and future directions

It is well recognized that the abnormal conditions in the tumor microenvironment affect the endothelial transcriptome [141–143]. As summarized here, the altered expression includes different galectins, predominantly galectin-1/-3/-8/-9. Moreover, the cellular localization, secretion and function of these endothelial galectins can be affected under malignant conditions. While different environmental triggers underlying the aberrant endothelial galectin expression have already been identified, it remains a challenge to unravel the mechanisms that control galectin expression and localization within the tumor vasculature. In addition, more insight in the mechanisms that regulate the endothelial glycome is necessary since alteration in protein glycosylation on the endothelial cell surface will affect the functional consequences of galectins [26, 67, 110]. Thus, further deciphering the galectin-glycan networks and linking these to endothelial cell activity remains an important future challenge.

Regarding the role of galectins in the tumor vasculature, the current literature predominantly suggests a tumor-promoting role by facilitation of both tumor angiogenesis and tumor immune escape. At the same time, there are still many unresolved questions regarding the exact mechanisms that underlie this angiostimulatory and immunosuppressive activity. Importantly, most studies have been performed using a single galectin or a specific immune cell type. Since the tumor microenvironment is far more complex, it can be anticipated that the activity of a given galectin on a given cell type is affected by the presence of other cells or other extracellular factors. For example, emerging evidence from us and others has shown that galectins can heterodimerize with other galectins, cytokines or growth factors, thereby controlling the activity of either protein [36, 39, 134, 144–146]. In addition, the expression and/or secretion of such molecules by different cell types within the tumor microenvironment will influence the ability of galectins to control endothelial or immune cell functionality. This is further complicated by the observation that galectins can show biphasic activity depending on the local concentration or activation status of the target cell [33, 114, 132, 147]. Deciphering such complex relationships remains challenging. In that regard, the development of animal models that allow conditional and (endothelial) cell specific knockdown of galectins or glycosylation pathways will be indispensable. Also, the current developments in (single cell) RNA sequencing as well as in spatial transcriptomics will help to uncover the role of (vascular) galectins the complex tumor microenvironment [148–150].

Despite the outstanding challenges, vascular galectins are considered as potential targets for cancer treatment. In fact, galectins are generally considered as potential therapeutic targets and many different galectin inhibitors have been developed, some of which have been tested in clinical trials (For a comprehensive recent review see [151]). Interestingly, endothelial cells are particularly attractive target cells since they are in direct contact with the blood and therefore easy reached by circulating drugs [3]. Moreover, targeting galectins in the vasculature could have dual benefit by simultaneously hampering tumor angiogenesis and alleviating immune suppression. This is supported by numerous studies showing that targeting galectins can hamper tumor progression by interfering with tumor vascularization and/or by stimulating an anti-tumor immune response [57, 140, 152–158]. For example, we recently published that vaccination against galectin-1 can increase tumor infiltration of cytotoxic CD8 + T cells resulting in reduced tumor growth [159]. Also recently, a novel set of orally available galectin-1 inhibitors was published that blocked the induction of T cell (Jurkat) apoptosis by galectin-1 [160]. Collectively, the continuous development of novel compounds and approaches to target galectins in combination with the unceasing efforts to unravel the mechanisms by which galectins contribute to tumor progression hold a promise for the treatment of future cancer patients. In particular since targeting endothelial galectins can have dual effects by inhibiting tumor angiogenesis and at the same time stimulating the anti-tumor immune response. This identifies galectins as interesting 'new kids on the therapeutic block'.

## Data Availability

Not applicable.
